# Supplementary material for: The apical ciliary adhesion complex is established at the basal foot of motile cilia and depends on the microtubule network
Source: Sci Rep. 2022 Nov 8;12:19028. doi: 10.1038/s41598-022-22871-0 (PMC9643470; doi:10.1038/s41598-022-22871-0)
Supplement: Supplementary file 1 — Supplementary Information 1. [file 41598_2022_22871_MOESM1_ESM.pdf]

The apical Ciliary Adhesion complex is established at the basal foot of motile cilia and depends on the microtubule network

Maria Chatzifrangkeskou and Paris A. Skourides

Supplementary files

Figure S1

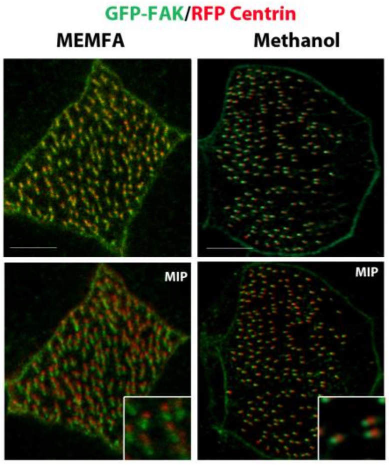

Figure S1: Confocal optical section or surface view (maximum intensity projection) of a MCC expressing GFP-FAK and RFP-Centrin fixed with MEMFA or methanol. Methanol fixation preserves apical CA localization.

Figure S2

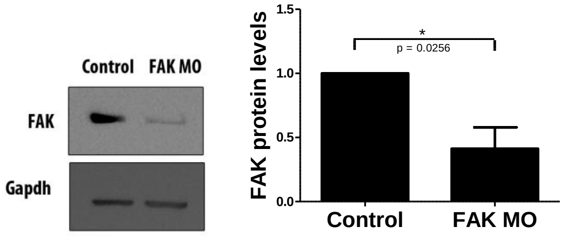

Figure S2: Western blot analysis of FAK protein levels in control embryos and FAK morphants. Quantification of FAK protein levels normalized to Gapdh. Error bars represent mean ± SEM from three independent experiments. Original blots are presented in Supplementary Information File.

Figure S3

A.

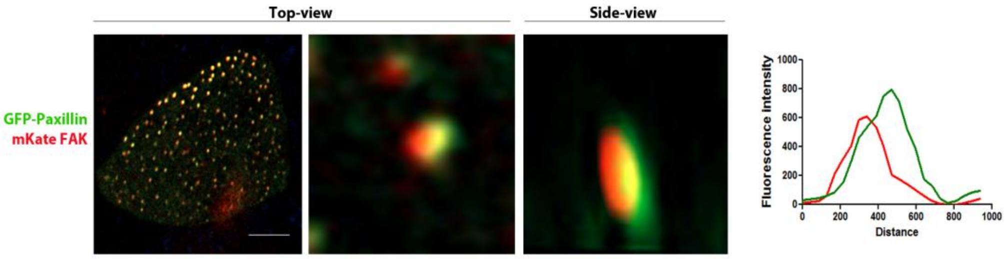

B.

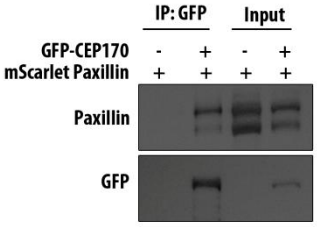

Figure S3: A) Confocal optical section of a multiciliated cell expressing GFP-Paxillin and mKate2-FAK. Scale bar represents 5 µm. B) Western blot showing immunoprecipitated GFP from HEK 293T cells co-transfected with GFP-CEP170 and mScarlet Paxillin, and blotted for GFP and Paxillin. Original blots are presented in Supplementary Information File.
