# Supplementary material for: The apical ciliary adhesion complex is established at the basal foot of motile cilia and depends on the microtubule network
Source: Sci Rep. 2022 Nov 8;12:19028. doi: 10.1038/s41598-022-22871-0 (PMC9643470; doi:10.1038/s41598-022-22871-0)
Supplement: Supplementary file 2 — Supplementary Information 2. [file 41598_2022_22871_MOESM2_ESM.pdf]

Supplementary Information File

Full-length western blots

Figure S2

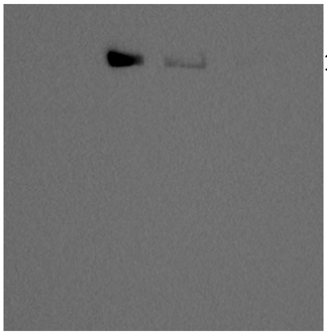

110 kDa

FAK

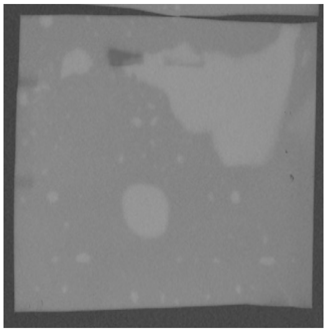

Membrane

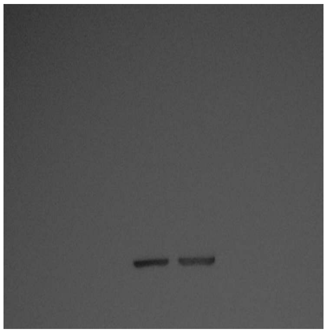

37 kDa

Gapdh

Replicates

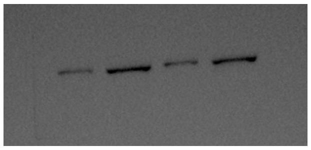

FAK

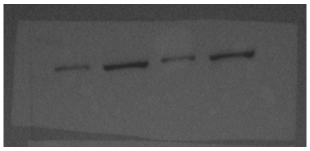

Membrane

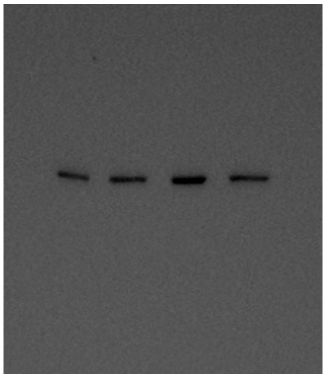

Gapdh

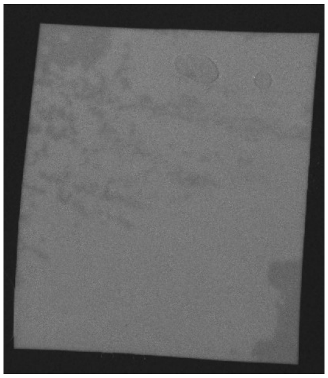

Membrane

Figure S3B

GFP

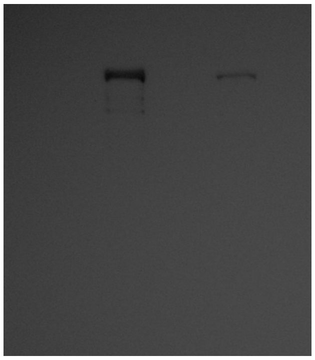

-200 kDa

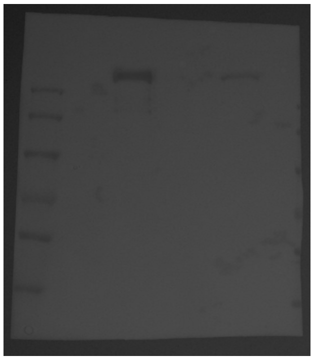

Membrane

Paxillin

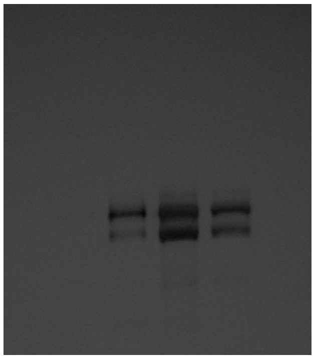

-95 kDa
-68 kDa
